# Group optimization methods for dose planning in tES

**DOI:** 10.1088/1741-2552/adf887

**Published:** 2025-08-14

**Authors:** R Salvador, J Zhou, B Manor, G Ruffini

**Affiliations:** 1 Neuroelectrics, Barcelona, Spain; 2 Harvard Medical School, Boston, MA, United States of America

**Keywords:** dose parameter, transcranial electrical stimulation, computational models

## Abstract

*Objective.* Optimizing transcranial electrical stimulation (tES) parameters—including stimulator settings and electrode placements, using magnetic resonance imaging-derived head models is essential for achieving precise electric field (E-field) distributions, enhancing therapeutic efficacy, and reducing inter-individual variability. However, the dependence on individually personalized MRI-based models limits their scalability in some clinical and research contexts. To overcome this limitation, we propose a novel group-level optimization framework employing multiple representative head models. *Approach.* The proposed optimization approach utilizes computational modeling based on multiple representative head models, selected to minimize group-level error compared to baseline (no stimulation). This method effectively balances focal stimulation intensity within targeted brain regions while minimizing off-target effects. We evaluated our method through computational modeling and leave-one-out cross-validation using data from 54 subjects, and analyzed the effectiveness, generalizability, and predictive utility of anatomical characteristics. *Main results.* Group-optimized protocols significantly outperformed standard template-based approaches when within-subject variability was accounted for using paired analyzes. Although average performance differences appeared modest in aggregate comparisons, paired statistical tests revealed that group-based solutions yielded systematically better targeting across participants. Additionally, group protocols consistently reduced the occurrence of poor outcomes observed with some templates. Correlations between anatomical features (e.g. head perimeter and tissue volumes) and E-field parameters revealed predictive relationships. This insight enables further optimization improvements through the strategic selection of representative head models that are electro-anatomically similar to the target subjects. Importantly, this approach eliminates the need for *a priori* selection of a single representative template, offering a scalable and more flexible alternative when individualized MRI-based models are not available. *Significance.* The proposed group optimization framework provides a scalable and robust alternative to personalized approaches, substantially enhancing the feasibility and accessibility of model-driven tES protocols in diverse clinical and research environments.

## 1. Introduction

The primary biophysical mechanism through which transcranial electrical stimulation (tES), including transcranial direct current stimulation (tDCS), interacts with neural tissues is the induced electric field (E-field), which drives the observed neuromodulatory effects. Thus, effective tES intervention critically depends on optimal dose planning, encompassing all controllable stimulator parameters (current amplitude and waveform), electrode positioning and geometry, and stimulation timing (Peterchev *et al* 2012). Early studies have highlighted the importance of carefully selecting these parameters to maximize therapeutic efficacy and minimize unwanted variability in stimulation outcomes (Nitsche and Paulus 2000, Miranda *et al* 2013, Saturnino *et al* 2015). With the rapid evolution in the field of image processing and the advent of powerful computational resources, several tools have become available to create volume conductor models that represent the passive electrical characteristics of different head tissues from structural images, usually T1w-MRIs (Datta *et al* 2009, Miranda *et al* 2013, Windhoff *et al* 2013). These biophysical head models were leveraged to optimize dose parameters such as electrode location and current, using optimization algorithms (Fernández-Corazza *et al* 2020, Dmochowski *et al* 2011, Janssen *et al* 2013, Ruffini *et al* 2014, Wagner *et al* 2016, Saturnino *et al* 2019b). Despite differences in the optimization function that these algorithms minimize (or maximize), and the methods that are used to optimize said function, all algorithms share common steps: they rely on pre-computed solutions of the E-field distribution (lead-field) induced using predetermined electrode positions; they assume some model for the interaction of the E-field with the neurons that determines the choice of optimization function; and they impose constraints on the currents for safety considerations and hardware specifications. Initial studies using these approaches typically relied on non-personalized head models based on template MRIs (Huang *et al* 2016, Zhou *et al* 2021). With advances in the processing pipelines, the use of head models personalized using subject-specific MRIs has become possible. This approach may improve stimulation effectiveness and lower its inter-subject variability (Salvador *et al* 2021). Studies employing personalized optimized protocols based on these algorithms have shown promising results in several recent trials (Aydin *et al* 2017, Kaye *et al* 2021, Daoud *et al* 2022). However, the practical implementation of personalized head modeling remains challenging in several scenarios. For instance, in clinical trials, MRIs may be unsuitable for creating accurate head models because of technical limitations, such as inappropriate acquisition sequences, severe motion-related artifacts, or excessive crops of the scalp, skull, and/or cerebrospinal fluid (CSF) tissues. In addition, obtaining new images can be constrained by timing or budgetary constraints. Furthermore, structural T1 image processing may fail in certain populations, including those with lesions, genetic malformations, and other structural anomalies. Personalized head models are also incompatible with studies that require the montage and protocol to be predefined before the participant imaging data becomes available.

Here, we propose a group optimization algorithm that improves upon single non-personalized head model optimizations in terms of the optimization metric, specifically, the proximity between the induced normal component of the E-field (*E_n_*-field) and the target *E_n_*-field distribution. The *E_n_*-field is considered particularly relevant because it represents the component of the E-field aligned with the cortical surface normal, which is thought to govern the modulation of pyramidal neuron excitability, key targets in tES. In this approach, instead of using a single subject/template for optimization, a representative sample of multiple subjects from the target population is used in the optimization, which should, on average, reduce the penalty in the objective function of the optimization resulting from a standard template model. We investigate this approach in an application targeting the lDLPFC, a region previously shown to benefit from tDCS-induced excitation in older adults, with improvements observed in dual-task performance during standing and walking (Zhou *et al* 2021). This target also aligns with recent and ongoing trials employing group-based stimulation strategies in aging and neurodegenerative populations. This target has been used in recent and ongoing trials employing group-based stimulation strategies in aging and neurodegenerative populations (see details below), as well as in studies on depression with positive results (Ruffini *et al* 2024).

## 2. Methods

### 2.1. Comparison plan

To study the performance of group optimization we compare normalized error with respect to no intervention (NERNI) goodness-of-fit of group-optimized montages in our cohort with personalized and *standard template* derived solutions. Additionally, we compared these approaches against an approach in which the protocol was derived from one of the subjects in the cohort (non-personalized solutions). Standard templates are commonly used to determine non-personalized protocols in many studies, as are head models obtained from subjects with similar demographic characteristics to the population of interest.

### 2.2. Head model creation

**Figure 1.**
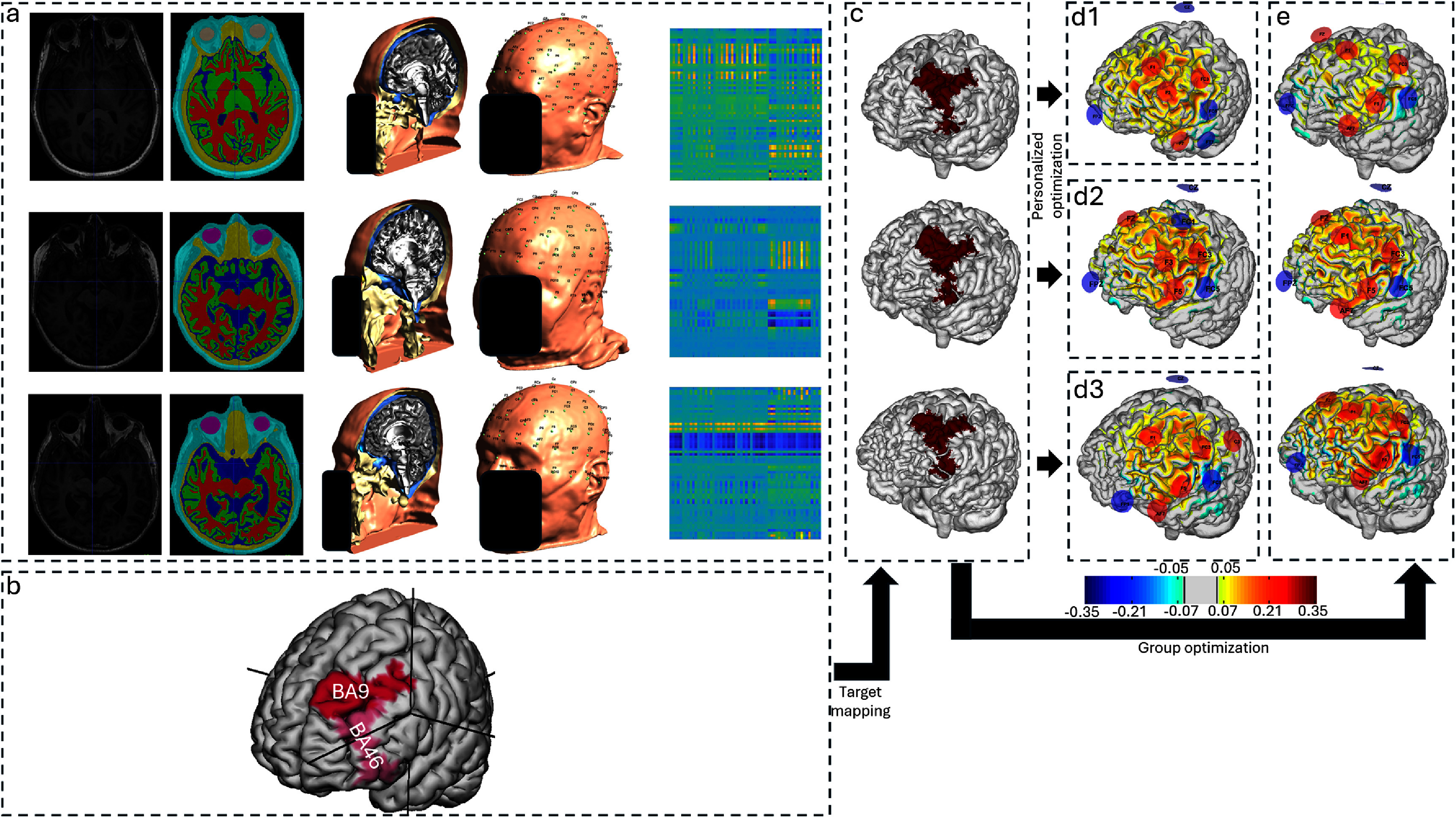
Overview of the modeling and optimization pipeline used in this study. (a) Left to right: structural head MRIs were segmented into major tissue types (skin, skull, cerebrospinal fluid, gray matter, and white matter); a tetrahedral finite element mesh was generated for electric field computation; 10–10 electroencephalography (EEG) system electrode positions were mapped to the surface of the scalp; and the lead-field matrix for the normal component of the electric field (${E_n}$) was calculated. Each column of the matrix represents the cortical *E_n_*-field induced by injecting 1 mA at a single electrode with Cz held as the fixed cathode. A 64 × 64 cropped section of the full matrix is shown for visualization clarity. The matrix visualization is provided as a qualitative illustration of the modeling process. (b) The stimulation target-the left dorsolateral prefrontal cortex (lDLPFC)- was defined in the head surface of the Montreal Neurological Institute (MNI) template as the union of Brodmann areas 9 and 46. (c) The lDLPFC target was then mapped to the gray-matter (GM) surface of each individual’s native head model. (d) For each participant, a personalized optimization was performed to maximize the electric field alignment with the target, using the normalized error with respect to no intervention (NERNI) as the cost function. The resulting ${E_n}$ field distribution is shown on each participant’s GM surface. (e) Same as in panel (d), but using a group-optimized protocol obtained by maximizing the average NERNI across all participants (leave-one-out approach). All ${E_n}$ maps are shown using a common color scale (in units of V m^−1^). All scalp surface reconstructions are anonymized.

We developed personalized head models from the structural MRI scans of 57 older adults (age ⩾ 65 years) who participated in one of two previous tDCS trials (NCT03814304 and NCT04295798). Both studies enrolled men and women aged 65 years or older, without overt neurological or psychiatric illness. All participants provided written informed consent as approved by the Hebrew SeniorLife Institutional Review Board. Each study was separately conducted in accordance with the principles of the Declaration of Helsinki and with all applicable local statutory requirements. T1w (GE scanner, 3D SPGR sequence, acquired with 32 channel head coil) and T2w (3D CUBE sequence) MRIs were segmented into the skin, skull, air cavities, CSF, gray matter (GM), and white matter (WM) using SimNibs (v3.2.1) (Saturnino *et al* 2019a) (figure 1(a), first column). All segmentations were inspected and corrected manually if deemed necessary. The most common corrections were due to errors in the CSF and bone over/under-segmentation. Upon inspection of all segmentations, one subject was removed from the analysis, because it showed a degree of brain atrophy that was considered an outlier compared to the other subjects in the group.

For each participant, a finite element mesh comprised of tetrahedra was built using SimNibs, and 10–10 EEG system positions were identified on the scalp surface (figure 1(a), second column). The positions were manually inspected for errors in the registration of the electrodes, which led to the removal of two more participants. The lead-field matrix for the E-field component perpendicular to the cortical surface (${E_n}$) was then calculated (figure 1(a), third column). Ag/AgCl electrodes, modeled as 1 cm radius cylinder of conductive gel, with a height of 3 mm were added to the head mesh, and tissues modeled as isotropic and homogeneous materials with specific conductivities (Mercadal *et al* 2022): 0.33 S m^−1^ for the skin, 0.008 S m^−1^ for the skull, 1.79 S m^−1^ for the CSF, 0.40 S m^−1^ for the GM, and 0.15 S m^−1^ for the WM. Air cavities were not meshed, resulting in an effective conductivity of 0 S m^−1^. The gel was modeled with a conductivity of 4.0 S m^−1^. Each column of the lead-field matrix (${\boldsymbol{K}}$) contains En per node (V m^−1^) for each bipolar montage with a fixed cathode (Cz, −1 mA). In accordance with SimNibs default settings, a Dirichlet boundary condition was used to fix the potential difference across the two electrodes. The electrodes current across the electrode surface was then calculated and the relative error with respect to the desired value (1.0 mA) was used to scale the solution. The finite element problems were solved using PETSc (https://petsc.org/main/manualpages/) with a conjugate gradient solver and a relative tolerance of 1 × 10^−10^. A BoomerAMG multigrid preconditioner from HYPRE was used with HMIS coarsening.

Similar methods were used to generate the head models for the templates Colin (Miranda *et al* 2013) and ICBM152 (ICBM 2009 c Nonlinear Asymmetric template) (Fonov *et al* 2011, Fonov *et al* 2009). All calculations were performed using custom Matlab (v2018b) scripts, which invoked SimNibs to solve Laplace’s equation and calculate the E-field distribution.

### 2.3. Montage and current optimization

The *Stimweaver* algorithm (Ruffini *et al* 2014) was used to maximize a fitness relative to no intervention (NERNI), defined as the least-squared difference between a weighted ${E_n}$ induced by the montage and a weighted target ${E_n}$ ($E_n^{{\text{trg}}}$),

\begin{align*}{\text{NERNI}} = \frac{{{{\left( {w \odot E_n^{{\text{trg}}}} \right)}^{\text{T}}}\left( {w \odot E_n^{{\text{trg}}}} \right) - {{\left( {{w^{\text{T}}}{\boldsymbol{K}}I - w \odot E_n^{{\text{trg}}}} \right)}^{\text{T}}}\left( {{w^{\text{T}}}{\boldsymbol{K}}I - w \odot E_n^{{\text{trg}}}} \right)}}{{{{\left( {w \odot E_n^{{\text{trg}}}} \right)}^{\text{T}}}\left( {w \odot E_n^{{\text{trg}}}} \right)}}\end{align*}

where $w$ is an array of weights (${N_{{\text{mesh}}}} \times 1$ array, where ${N_{{\text{mesh}}}}$ is the number of mesh nodes in the cortical GM surface), $E_n^{{\text{trg}}}$ is the target ${E_n}$-field array (dimensions of ${N_{{\text{mesh}}}} \times 1$, in units of V m^−1^), ${\boldsymbol{K}}$ is the lead field matrix (${N_{{\text{mesh}}}} \times {N_{{\text{electrodes}}}} - 1$, in units of V m^−1^ per mA of injected current, ${N_{{\text{electrodes}}}}$ is the number of electrode positions defined in the scalp), and $I$ is the array with electrode currents in mA (Cz excluded, as its current is defined implicitly). $ \odot $ represents the Hadamard symbol, i.e. the element-wise multiplication of arrays/matrices of similar dimensions.

NERNI is a scalar fit parameter that measures the similarity of ${E_n}$–$E_{_n}^{{\text{trg}}}$. It is equal to one for a perfect fit and a large negative number when the fit is poor.

Targets were defined by mapping Brodmann areas 9 and 46 (lDLPFC) from an MNI template to the GM surface of each participant (figure 1(b)). The mapped targets for all subjects, illustrating inter-subject variability and consistency in anatomical location, are provided in the supplementary material. The optimization constrained the maximum current at any electrode to 2.0 mA and the total injected current to 4.0 mA using a genetic algorithm to limit the montage to eight electrodes. No other constraints were imposed on the polarity or values of the currents per electrode. There were no explicit constraints imposed on the polarity (number of anodes versus cathodes). However, the optimization was limited to a maximum of eight electrodes, with the additional constraint that the sum of currents across all electrodes equaled 0 mA (balanced current constraint). This allowed the optimizer flexibility in assigning currents and polarities to electrodes, subject to these overall constraints. The target ${E_n}$ was set to 0.25 V m^−1^ in the lDLPFC, with a weight of 10 indicating an increase in cortical excitability according to the lambda-E model for interactions of the E-field with neurons (Ruffini *et al* 2014). This target ${E_n}$ was selected empirically based on prior work using similar modeling pipelines. It reflects a balance between achieving sufficient intensity to reach the target threshold for excitability and maintaining focality; in practice, we observed that this target level typically leads to use of the maximum available injected current. Higher target values may result in stronger fields but at the cost of broader stimulation spread. In this model, the effects of stimulation on cortical excitability are due to the interaction of the normal component of the E-field with large pyramidal cells in the cortex. When ${E_n}$ points to the cortical surface (positive ${E_n}$ in the convention followed in this work), the soma of pyramidal cells is depolarized, which leads to an increase in cortical excitability. The negative ${E_n}$ values have the opposite effect. This model appears to be representative of the effects of weak E-fields on neuronal membranes (Galan-Gadea *et al* 2023). In the other areas, the target ${E_n}$ was set to 0 V m^−1^ (no effect), with a lower weight of 2.

This weighting scheme was chosen to balance focality and field strength in the target region. Assigning equal weights across all mesh nodes would result in the much larger off-target area dominating the optimization, leading to overly focal montages that may produce insufficient field intensity at the target (i.e. underdosing). By assigning a higher weight to the target region, the optimization prioritizes delivering sufficient *E*_n_ while still penalizing field spread to non-target areas. This trade-off was informed by prior internal analyses and is consistent with retrospective studies using similar modeling frameworks (e.g. Salvador *et al* 2022).

The calculation of the normal vector per mesh node was done by considering the weighted average of the normal to each triangle that contains the node, with the weights being the area of the triangle.

Optimization was conducted using a genetic algorithm, as explained in more detail by Ruffini *et al* (2014). Initially, a population (*N* = 3000) representing combinations of electrode positions was created randomly. Then, for each combination (DNA string), SLSQP (sequential least squares programming optimization) was used to determine the currents that maximized the NERNI. The best members of the population were then selected, and genetic operators were used to determine the next population. This process was repeated and stopped when the improvement in the best NERNI was less than a specified threshold (10^−6^) for five consecutive generations of the genetic algorithm. These parameters led to a solution that was consistently within 99% of the NERNI of the solution with an unlimited number of electrodes. The method was implemented in Python, using DEAP and SciPy as the main libraries. On a standard workstation, single-subject optimizations completed in under 5 min, while group optimizations (across 53 participants) took approximately 5 h due to higher computational demands.

Protocol optimization was performed for the participants individually (figure 1(d1–3)). Additionally, group optimizations (figure 1(e)) were conducted using a leave-one-out approach: for every participant a group optimization was conducted using the remaining 53 participants (templates excluded). The group optimization is similar to the single participant optimization, but the objective function is the arithmetic mean of NERNI for each of the participants included in the group (${N_{sg}}$),

\begin{align*}{\text{NERN}}{{\text{I}}_g} = \frac{1}{{{N_{sg}}}}\mathop \sum \limits_{s = 1}^{{N_{sg}}} \frac{{{{\left( {{w_s} \odot E_{n,s}^{{\text{trg}}}} \right)}^{\text{T}}}\left( {{w_s} \odot E_{n,s}^{{\text{trg}}}} \right) - {{\left( {{w_s}^{\text{T}}{{\boldsymbol{K}}_{\boldsymbol{s}}}I - {w_s} \odot E_{n,s}^{{\text{trg}}}} \right)}^{\text{T}}}\left( {{w_s}^{\text{T}}{{\boldsymbol{K}}_{\boldsymbol{s}}}I - {w_s} \odot E_{n,s}^{{\text{trg}}}} \right)}}{{{{\left( {{w_s} \odot E_{n,s}^{{\text{trg}}}} \right)}^{\text{T}}}\left( {{w_s} \odot E_{n,s}^{{\text{trg}}}} \right)}}.\end{align*}

Similar methods were used to maximize ${\text{NERN}}{{\text{I}}_g}$. It should be noted that all group-optimized protocols are different, since the subject pool varies from case to case.

An additional metric was used to evaluate the performance of the montage, the surface average of ${E_n}$ ($\left\langle {{E_n}} \right\rangle $),

\begin{equation*}\langle {E_n}\rangle = \frac{{{{\mathop \sum \nolimits}}{{}}_{i = 1}^{{N_{{\text{target}}}}}{E_{n,i}}{A_i}{ }}}{{{{\mathop \sum \nolimits}}{{}}_{i = 1}^{{N_{{\text{target}}}}}{A_i}{ }}}.\end{equation*}

Here, ${N_{{\text{target}}}}$ is the number of nodes in the main target region (lDLPFC) in the GM surface, ${E_{n,i}}$ is the ${E_n}$ induced at node $i$, and ${A_i}$ is the area of the node (the sum of the areas of all triangles that share the node, divided by 3). We included $\left\langle {{E_n}} \right\rangle $ as a secondary metric because it is more intuitive and easily interpretable than NERNI, being commonly used in tES literature (Van Hoornweder *et al* 2023). It also offers a complementary check to ensure that protocols achieving good NERNI scores are not doing so by minimizing off-target error alone but are also producing substantial field in the target.

### 2.4. Calculation of anatomical features

To test whether the anatomical features of the head models were associated with the NERNI of a protocol, we calculated several global anatomical features,

• Axial perimeter: geodesic distance along the scalp between Nz-LPA-Iz-RPA (L/RPA: left/right pre-auricular points) in mm
• Sagittal perimeter: geodesic distance along the scalp between Nz-Cz-Iz (mm)
• Coronal distance: geodesic distance along the scalp between LPA-Cz-RPA (mm)
• Volumes of tissues: volumes of the scalp, skull, CSF (excluding ventricles), GM and WM in mm^3^

**Figure 2.**
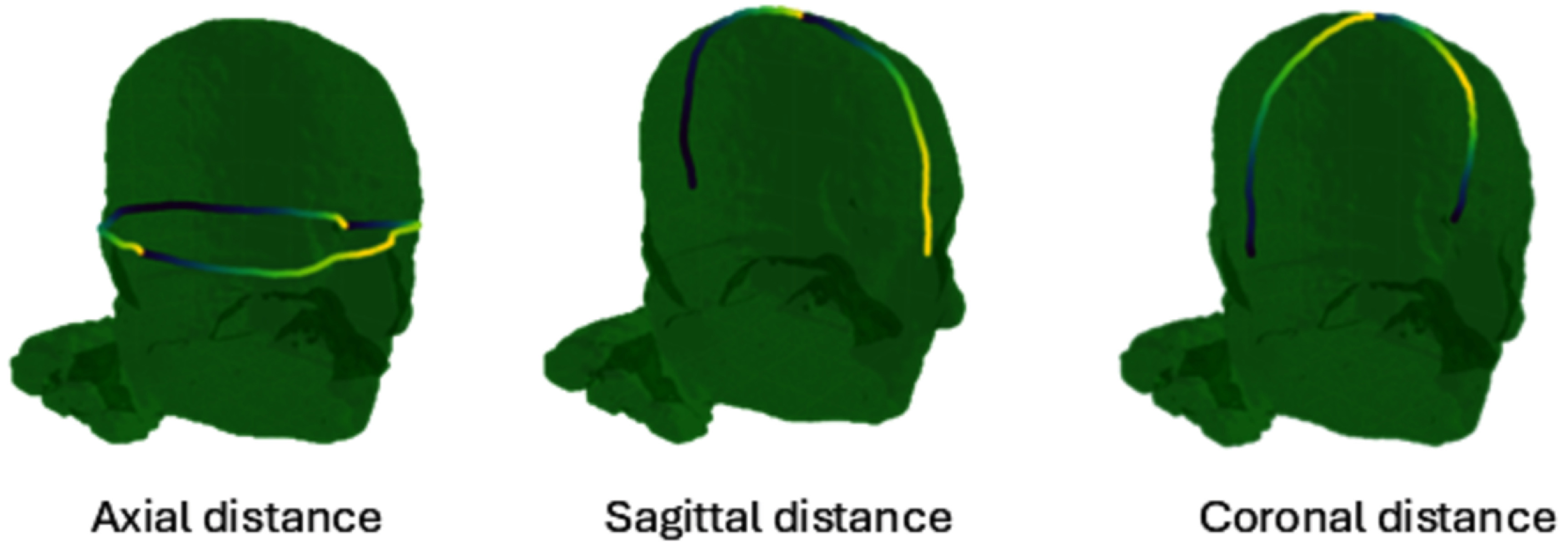
Scalp reference distances measured along the scalp of one participant. The different curved lines represent geodesic distances between standard anatomical landmarks commonly used in the electroencephalography (EEG) 10–10 system: nasion (Nz), inion (Iz), left and right preauricular points (LPA and RPA). These paths were used to compute reference measurements such as head perimeters in the axial, sagittal, and coronal planes for subsequent anatomical feature analyzes.

Figure 2 illustrates the measurement of distances for one participant. The distance features can be assessed without the need for MRI (these distances can be obtained from the head of the participant with a measuring tape). The volume features require an MRI and at least a segmentation of the different tissues. The distances were measured in *Python* with the *Pygeodesic* library, using the triangulated scalp surface of each participant. To calculate the volumes, the *MSH* files with the finite element meshes of each participant were loaded into *MATLAB* (v2018a). The volume of each tetrahedron comprising each tissue was then calculated and summed.

### 2.5. Statistical tests

All statistical tests were performed in *Python* (v3.7.9) with in-house functions that used *Sklearn* and *Scipy Python* libraries. Multilinear regression models were performed using with the *Statsmodel.api* library. For group comparisons of NERNI scores across protocol types (e.g. personalized, group-optimized, template-based), we initially employed a nonparametric Kruskal–Wallis test, followed by post-hoc pairwise comparisons using the Dunn test with Bonferroni correction. While this approach is robust to non-normality, it does not account for within-subject dependencies.

To more appropriately reflect the repeated-measures nature of our design (i.e. each subject evaluated under all protocol types), we also conducted pairwise paired *t*-tests between protocol conditions. These comparisons were adjusted for multiple testing using the Bonferroni method, and the corrected *p*-values were used to annotate significance in the visualizations. Paired *t*-tests were implemented using scipy.stats.ttest_rel, and *p*-value adjustments were carried out with statsmodels.stats.multitest.multipletests.

Regression analyzes (e.g. predicting $\left\langle {{E_n}} \right\rangle $ or NERNI from anatomical features) were evaluated using linear and multilinear regression models. In these cases, *p*-values reported in the Results section correspond to *F*-tests assessing the significance of the overall regression model, that is, whether the model explains a statistically significant portion of variance in the outcome (null hypothesis: *R*^2^ = 0). Where applicable, we used cross-validation (e.g. leave-one-subject-out) to assess model generalizability. Comparisons between regression models (e.g. with or without PCA, or using different anatomical features) were based on differences in *R*^2^ values and are reported descriptively.

## 3. Results

### 3.1. Personalized and group montages perform better than templates

**Figure 3.**
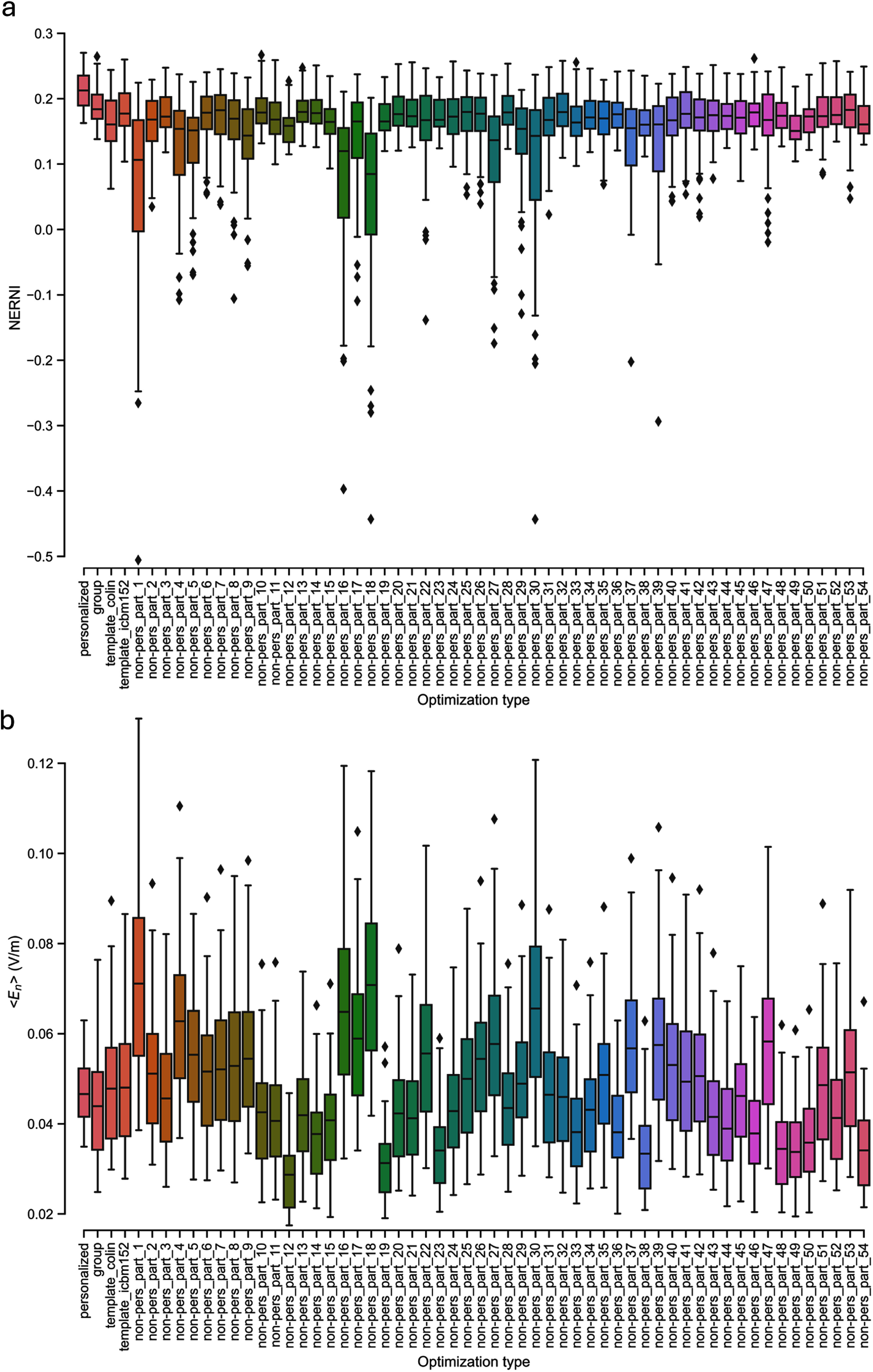
Distributions of targeting outcomes across 64 participants, grouped by optimization type. (a) Distribution of the normalized error with respect to no intervention (NERNI), a measure of how closely the induced normal component of the electric field (${E_n}$) matches the target map. (b) Distribution of the surface average of ${E_n}$ in the target region ($\left\langle {{E_n}} \right\rangle $). Each distribution corresponds to a different optimization approach: personalized (subject-specific), group-optimized, Colin-based template, ICBM152-based template, and templates derived from individual biophysical head models within the cohort (labeled as non-pers_part_<*id*>).

Across all participants (see figure 3(a)), the personalized protocol performed best in terms of fit (NERNI =0.21 ± 0.03, average ± standard deviation across all participants). Both head model templates (Colin and ICBM152) performed worse than the personalized approach: 0.16 ± 0.03 and 0.18 ± 0.03, respectively, for Colin and ICBM152. The group approach performed better than the templates but worse than the personalized protocol: 0.19 ± 0.03.

**Figure 4.**
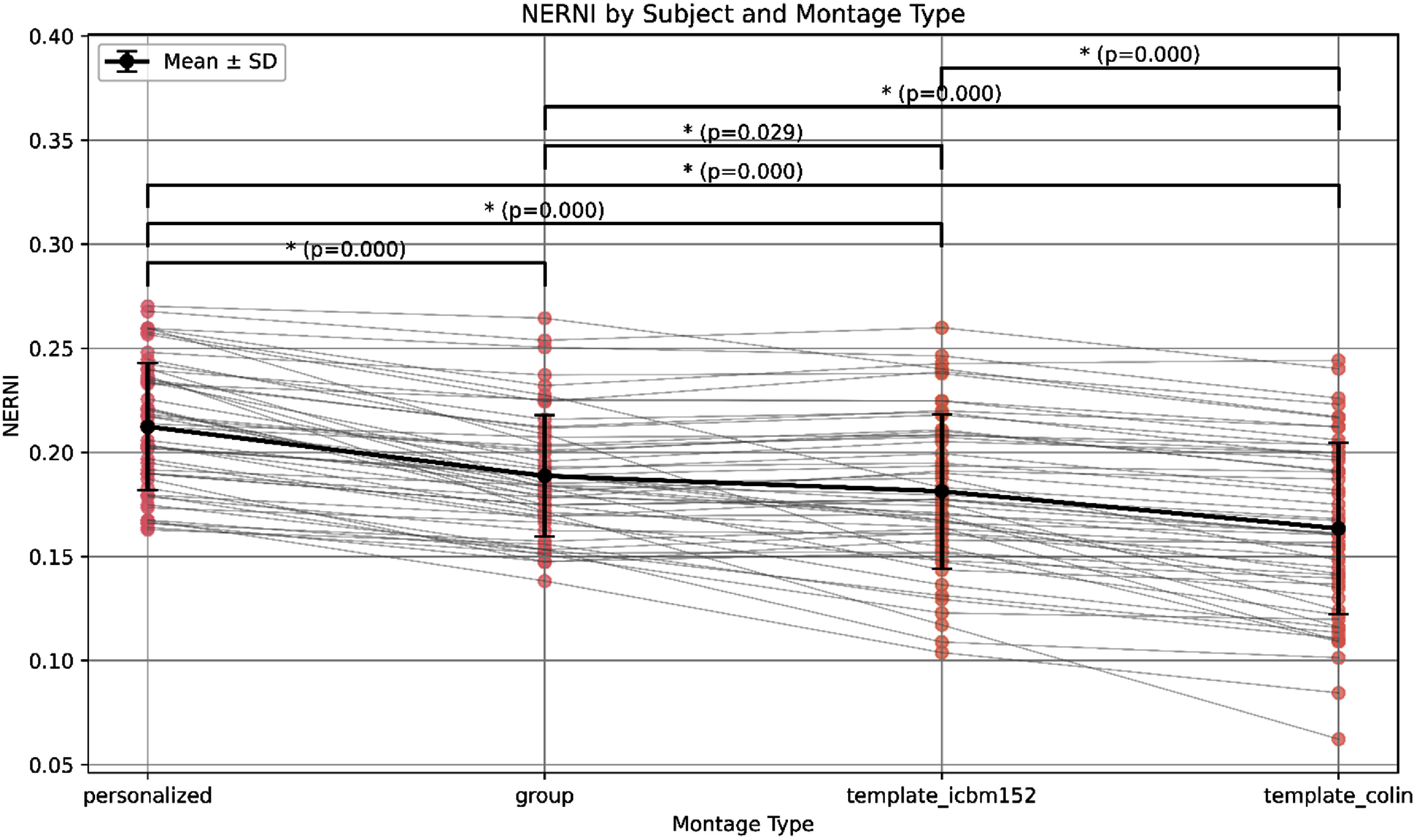
Normalized error with respect to no intervention (NERNI) values across 54 participants for each optimization approach. Gray lines show within-subject changes across montage types, while black circles with error bars indicate the group mean ± standard deviation. Statistical comparisons were conducted using two approaches: (1) a nonparametric Kruskal–Wallis test followed by post-hoc Dunn tests with Bonferroni correction, and (2) paired *t*-tests accounting for the repeated-measures design, also corrected for multiple comparisons. Annotated *p*-values reflect results from the paired *t-*tests. Asterisks denote statistically significant differences (*p* < 0.05, Bonferroni-corrected).

Initial comparisons using the Kruskal–Wallis test and Dunn post-hoc analyzes revealed significant differences between the personalized protocol and all other conditions, as well as between the group and Colin templates. No significant difference was found between the group and ICBM152 protocols using this method. However, given the within-subject design of the experiment, we performed follow-up paired *t*-tests to better capture individual-level differences across conditions. These revealed that the group-optimized montage significantly outperformed both template-based protocols, including ICBM152 (*p* < 0.05, Bonferroni-corrected). These additional comparisons, which account for intra-subject variability, are reflected in the updated annotations in figure 4. Notably, the group approach also yielded fewer extreme low-NERNI outcomes across subjects, supporting its robustness and potential as a viable alternative when personalized modeling is not feasible.

**Figure 5.**
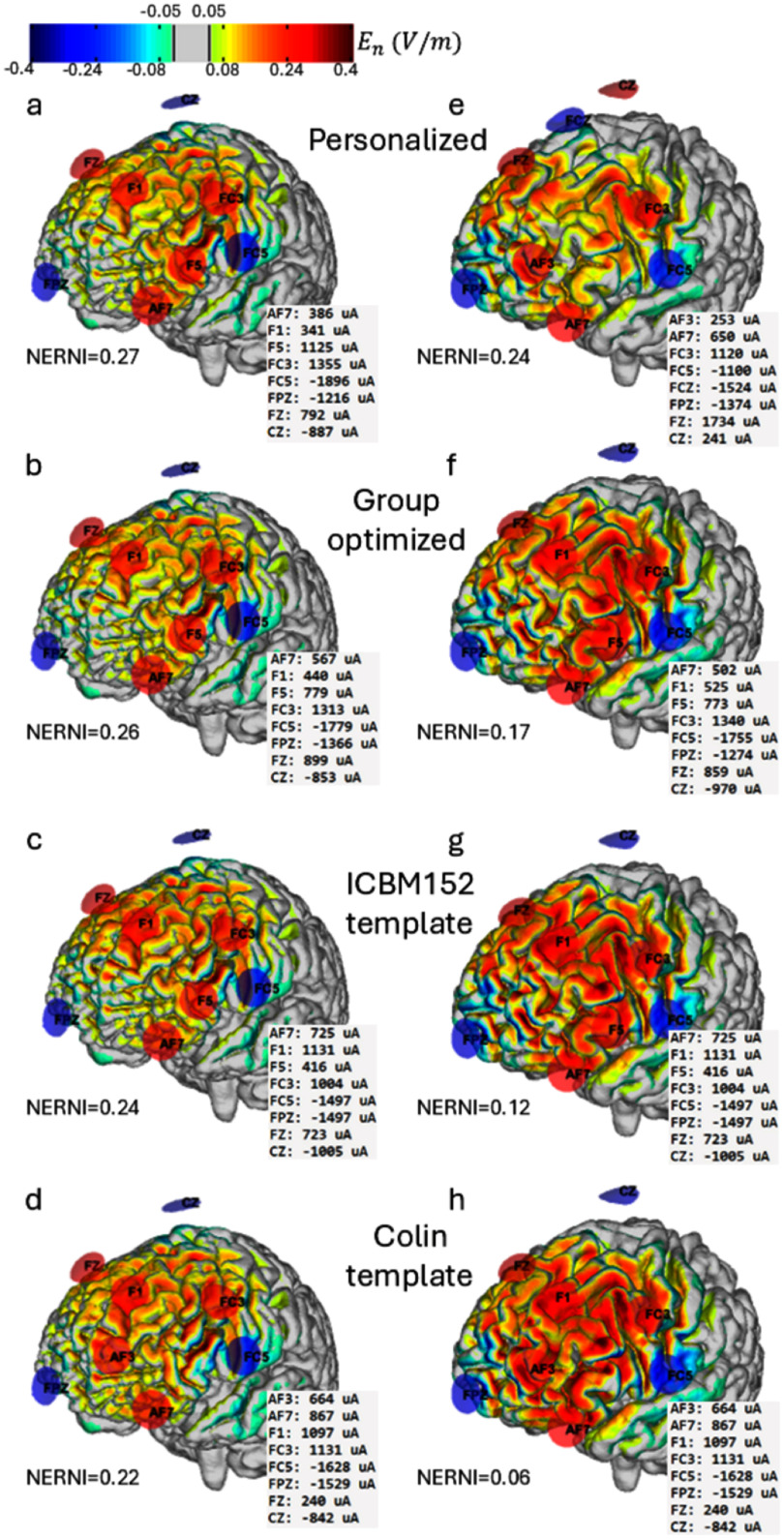
Distribution of the normal component of the electric field (${E_n}$) on the cortical surface for two participants: one in whom the group-optimized protocol achieved the best fit (left column) and one in whom it performed the worst (right column), based on the normalized error with respect to no intervention (NERNI). For each participant, the panels (top to bottom) show the results from: the personalized protocol, the group-optimized protocol (leave-one-out approach), the protocol obtained with the ICBM152-based template, and the protocol obtained with the Colin-based template. All ${E_n}$ distributions are visualized using a common color scale (in V m^−1^).

An example of these differences can be seen in figure 5, for the case where the group approach performed the best (figures 5(a)–(d)) and for the case where it performed the worst (figures 5(e)–(h)). It can be seen that the protocols that result in the lower NERNI have a less focal *E*_n_-field distribution, resulting in a worse fit to the target map.

## 4. Using arbitrary individual models vs. standard templates

Using a non-personalized template derived from the head model of one participant led to variable results, as shown in figure 3(a). When compared to other approaches, these non-personalized templates always performed worse than the personalized templates (*p*-value < 0.05). The group-optimized approach consistently resulted in higher NERNI average scores than the non-personalized templates, with statistically significant differences in 38 of the 54 participants. The ICBM152 template yielded higher NERNI values than the non-personalized templates in 48 out of 54 participants (89%), with 24 of those differences reaching statistical significance. The Colin template showed higher NERNI in 20 out of 54 cases (37%), with nine statistically significant differences.

**Figure 6.**
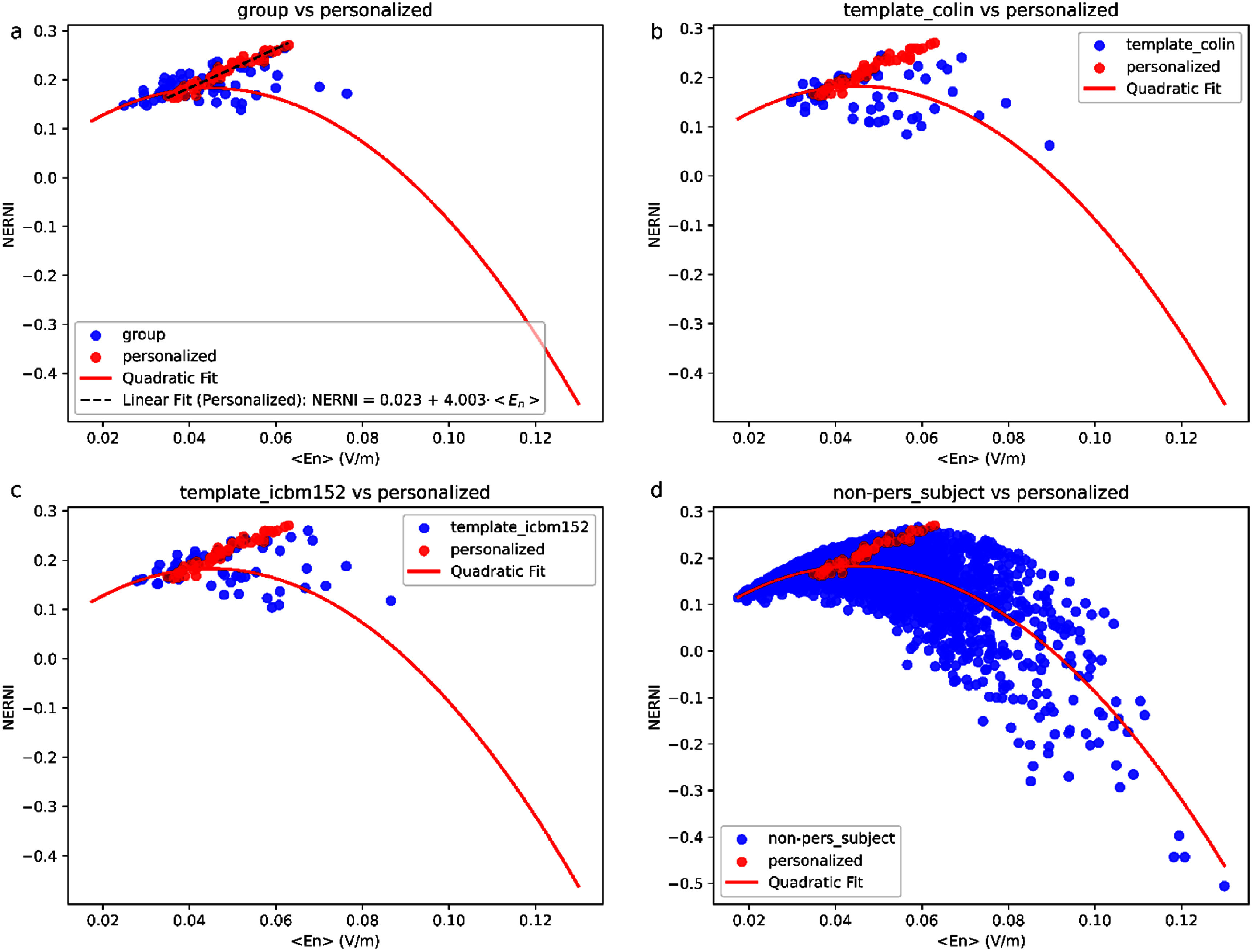
Relationship between the normalized error with respect to no intervention (NERNI) and the surface average of the normal component of the electric field $\left\langle {{E_n}} \right\rangle $ in the left dorsolateral prefrontal cortex (lDLPFC) across 64 participants. Each subplot compares one optimization approach (blue) with the personalized approach (red): (a) group-optimized, (b) Colin-based template, (c) ICBM152-based template, and (d) templates derived from individual biophysical head models within the cohort. The solid red line shows a quadratic fit to the combined dataset across all conditions, given by: NERNI = 0.003 + 7.999·$\left\langle {{E_n}} \right\rangle $ −89.125·$ &lt; {E_n}{ &gt; ^2}$. In panel (a), a dashed black line indicates the linear fit for the personalized data: this illustrates the tighter and more linear relationship observed in the personalized optimizations.

When examining the distribution of the $\left\langle {{E_n}} \right\rangle $ in the target region, we can see that there are considerable fluctuations in the average value, depending on the approach used to generate the protocol (figure 3(b)). A larger $\left\langle {{E_n}} \right\rangle $ on target did not predict good performance for the NERNI. For instance, the montages from participants 1, 16, and 18 had a large average $\left\langle {{E_n}} \right\rangle $ when evaluated in the other participants (albeit with larger variability as well), but the average NERNI was among the lowest. The relationship between NERNI and $\left\langle {{E_n}} \right\rangle $, shown in figure 6, shows that the entire data combination is well fitted by a quadratic regression model (*R*^2^ = 0.46, with a *p*-value of fit coefficients of 0.567 for the intercept, non-significant, but highly significant for the first and second-order terms: *p*-value < 0.001). This indicates that very low and very high $\left\langle {{E_n}} \right\rangle $ values result in poor NERNI. The personalized approach restrains the protocols in a region where there is a linear increase in NERNI with $\left\langle {{E_n}} \right\rangle $ (figure 6(a)). The group approach, ICBM152, and Colin template-derived protocols performed increasingly worse, as shown by the larger spread in $\left\langle {{E_n}} \right\rangle $ and NERNI values (figures 6(a)–(c)). The non-personalized templates derived from the head models of the participants resulted in a large spread of $\left\langle {{E_n}} \right\rangle $ and NERNI (figure 6(d)).

### 4.1. Anatomical features drive variance in template average En and NERNI

For the data derived using non-personalized templates obtained from the head models of the participants, we investigated the correlation of differences in anatomical features between the template head model and those of the participants in which the template was evaluated. These regression analyzes linking anatomical features to NERNI and ${E_n}$ were performed as an exploratory investigation into whether simple, low-burden anatomical measurements could predict targeting performance. Although NERNI and ${E_n}$ reflect complex, nonlinear outcomes of optimization, correlations with anatomical differences would support the feasibility of using such measurements to guide subject selection for group optimization. This would help ensure that participants included in a group optimization pool are more anatomically similar to the target individual, potentially improving targeting success without requiring full MRI-based modeling.

**Figure 7.**
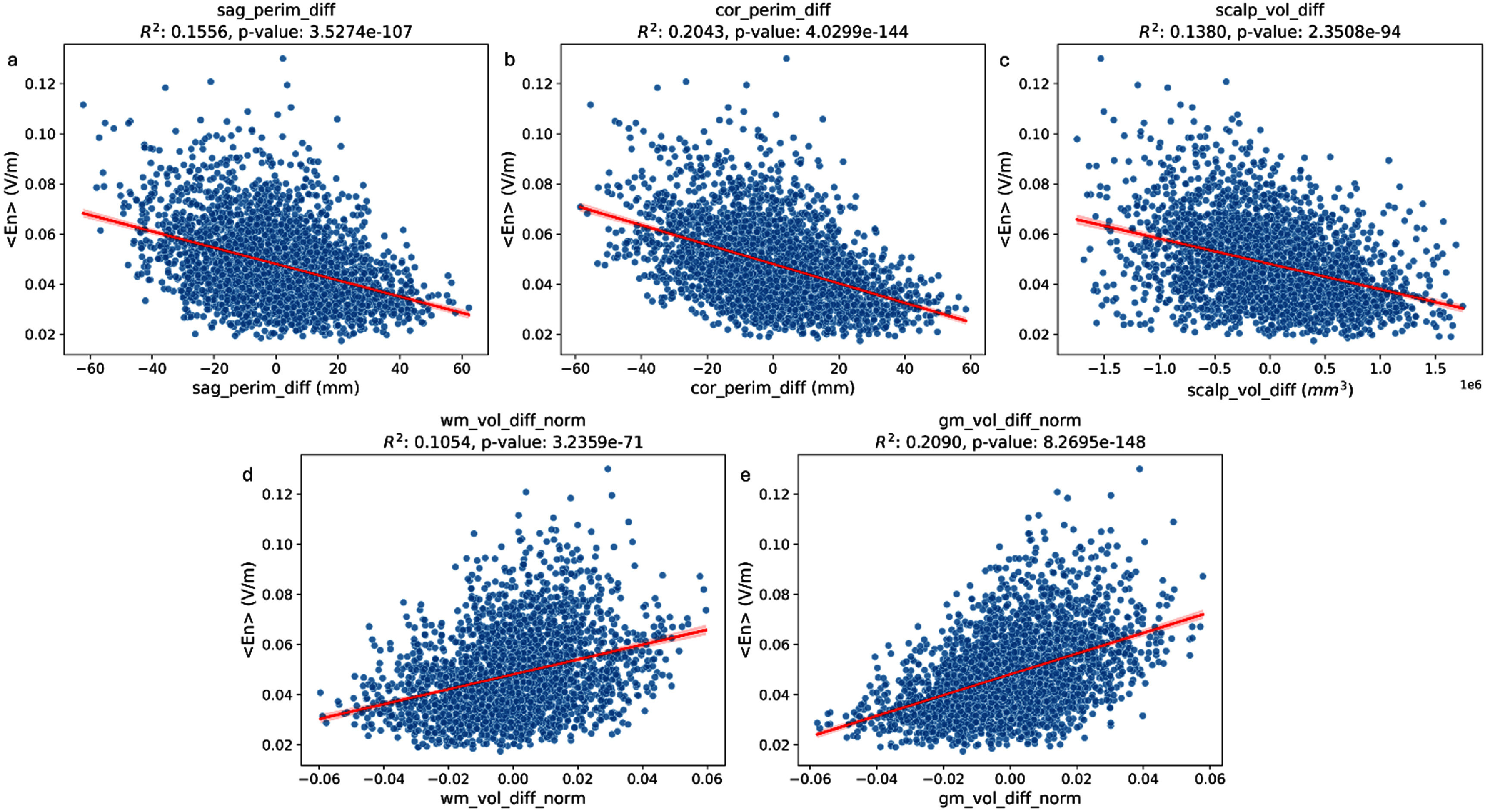
Linear regressions between the surface average of the normal component of the electric field on target ($\left\langle {{E_n}} \right\rangle $) and differences in anatomical features between each participant and the template used to evaluate the protocol (i.e. a non-personalized template, based on the biophysical head model of one of the other participants). From left to right, the regressors are: (i) difference in head perimeter along the sagittal and coronal planes (sag/cor_perim_diff, a/b), (ii) difference in scalp volume (scalp_vol_diff, c), and (iii) difference in normalized gray and white matter volumes computed by dividing by the total head tissue volume (WM/GM _vol_diff_norm, d/e).

Linear regressions of $\left\langle {{E_n}} \right\rangle $ against each tested individual feature showed that some of them significantly explained a small amount of the variability in NERNI (*p*-value < 0.05). The features with the highest correlation (see figure 7) with $\left\langle {{E_n}} \right\rangle $ were the differences in the perimeter along the sagittal (${R^2} = 0.16$) and coronal planes (${R^2} = 0.20$), the difference in scalp volume (${R^2} = 0.14$), and the difference in WM and GM volume, normalized by the sum of the volumes of all tissues (${R^2} = 0.11$ and ${R^2} = 0.21$).

**Figure 8.**
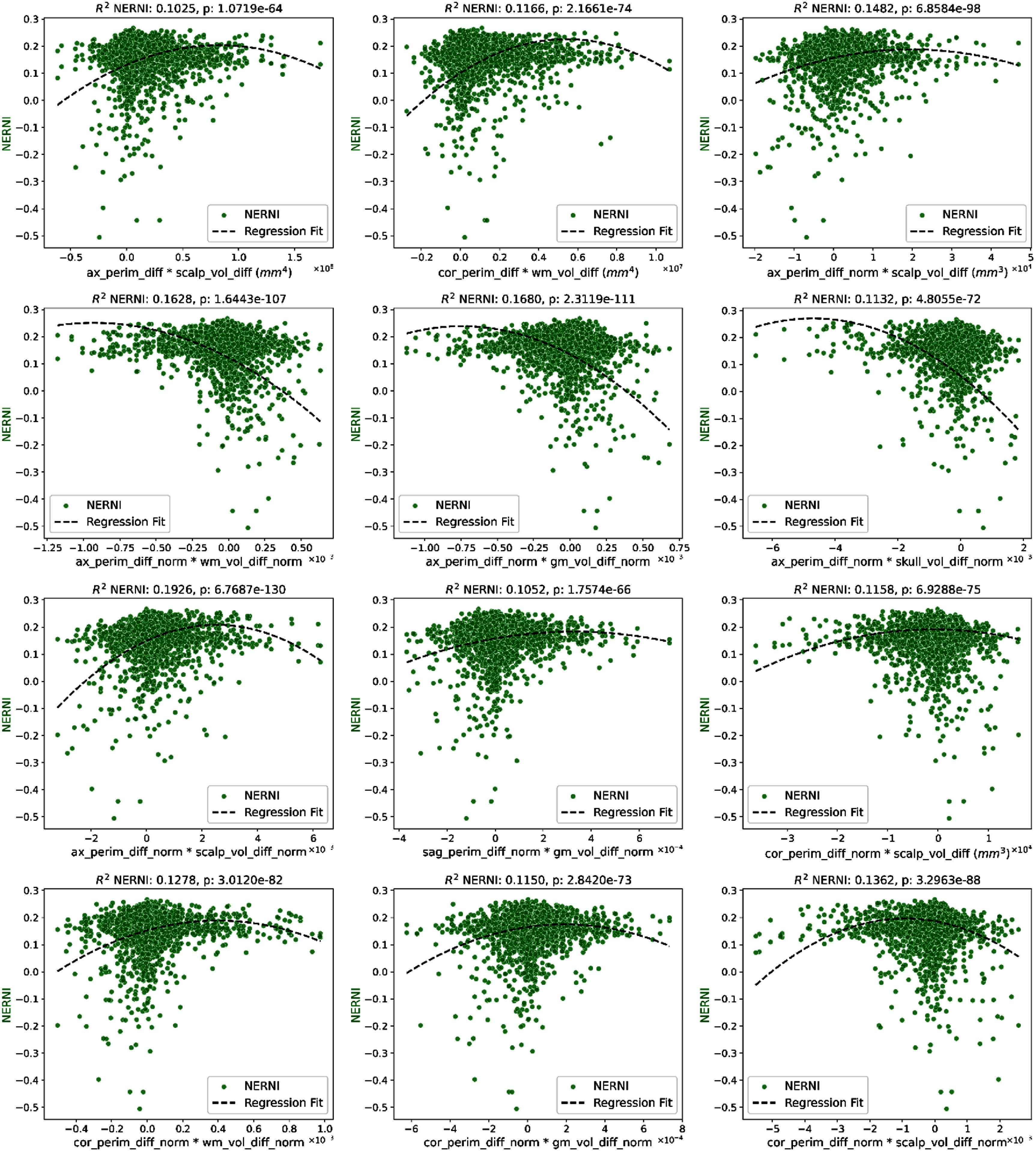
Linear regression results predicting the normalized error with respect to no intervention (NERNI) using anatomical features between participants and the non-personalized templates used to evaluate each protocol. The regressors include all first- and second-order interaction terms derived from combinations of anatomical features. Only models with ${R^2}$ values larger than 0.1 are shown. Relevant features include: ax/cor_perim_diff (difference in scalp perimeter measured along the axial and coronal planes), ax/cor/sag_perim_diff (combined perimeter differences in axial, coronal and sagittal planes, normalized by the sum of all 3 perimeters), scalp/wm_vol_diff (difference in scalp and white matter volume), and scalp/wm/gm/skull_vol_diff_norm (differences in scalp, white matter, gray matter, and skull volume normalized by total tissue volume).

To perform the same analysis to NERNI, and since the previous results showed that NERNI could be fit to $\left\langle {{E_n}} \right\rangle $ values using a 2nd order polynomial, we used a linear regression model taking as features all second-order terms in every possible combination of two features (feature-1, feature-2, feature-1 × feature-2, feature-1^2^ and feature-2^2^) resulting in the plots shown in figure 8 (only correlations with *R*^2^ larger than 0.1 are shown). The interactions that explain most of the variability in the data typically involve a perimeter feature (the differences in axial/sagittal/coronal distance, normalized by the sum of all three distances) and a volume feature (the difference in the scalp, skull, GM, WM, normalized, or not, by the sum of all volumes).

Since several of these features correlated with $\left\langle {{E_n}} \right\rangle $ /NERNI, we explored multilinear regression models to assess their predictive power. To evaluate the practical applicability of these models in a realistic scenario, we performed a leave-one-subject-out approach: for each subject, we removed all data from that individual, fit the multilinear regression model using the remaining participants, and then predicted the average $\left\langle {{E_n}} \right\rangle $/NERNI induced by the subject’s personalized protocol in the other participants. Importantly, these predictions are based only on anatomical features.

**Figure 9.**
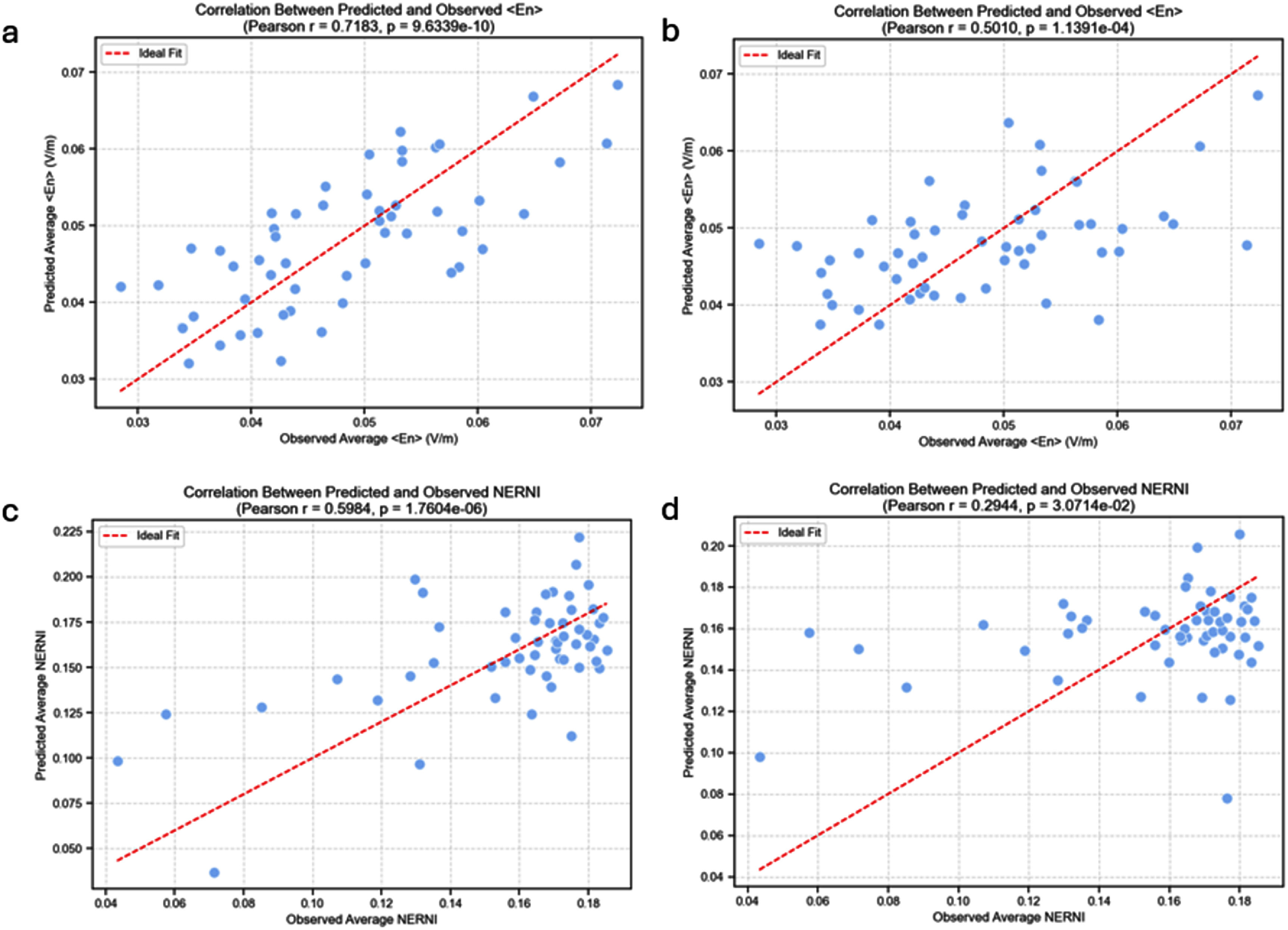
Predicted versus observed values of the surface average of the normal component of the electric field on the target region ($\left\langle {{E_n}} \right\rangle $, top row) and the normalized error with respect to no intervention (NERNI, bottom row), obtained through leave-one-subject-out cross-validation. For each participant, a regression model was trained using the anatomical features and NERNI/$\left\langle {{E_n}} \right\rangle $ values from the remaining *N*−1 participants and used to predict that participant’s value. (a/c) Models include all anatomical features (scalp perimeters and tissue volumes); (b/d) models include only perimeter-derived features. Each point represents one participant. The diagonal line indicates perfect prediction.

This approach resulted in a strong correlation with the observed $\left\langle {{E_n}} \right\rangle $ values (${R^2} = 0.52$, *p*-value < 0.001), as shown in figure 9(a). While some of the anatomical features included in the previous analysis require MRI-based segmentation, others, such as the perimeters along the axial, sagittal, and coronal planes, can be measured without MRI access. When restricting the regression model to only these perimeter-derived features, we still found a statistically significant correlation with the observed $\left\langle {{E_n}} \right\rangle $ values (${R^2} = 0.25$, *p*-value < 0.001; figure 9(b)).

Extending the model to include second-order combinations of anatomical features was also successful in predicting NERNI values for both the model with the tissue volume features (figure 9(c), ${R^2} = 0.36$, *p*-value < 0.001) and that with only the perimeter-derived features (figure 9(d), ${R^2} = 0.09$, *p*-value = 3.1 × 10^−2^). Because there is a high collinearity between some of these features, we also tried an approach where PCA was performed before regression. This was done to reduce multicollinearity and stabilize the regression coefficients, rather than to gain interpretability. The model was then fit using a subset of the principal components selected via cross-validation. This resulted in slightly better results: ${R^2} = 0.43$, *p*-value < 0.001 (model with all the features, and keeping 97 principle components for the fit); ${R^2} = 0.09$, *p*-value = 2.6 × 10^−2^ (model with only perimeters, and keeping 17 components).

## 5. Discussion

Our results indicate that protocol optimization algorithms can benefit from group-based approaches instead of relying on a single head model in settings where personalized head models cannot be obtained. On average, the group approach produced NERNI scores that were consistently higher than those obtained when using a particular head template, either standard templates (such as those derived from Colin or ICBM152) or templates obtained from the same cohort. While initial nonparametric analyzes did not detect statistically significant differences between the group and ICBM152 protocols, subsequent paired *t*-tests (accounting for the repeated-measures design) revealed that the group approach significantly outperformed both standard templates. Additionally, group-based protocols consistently avoided the lowest NERNI outliers observed with template-based approaches, suggesting improved robustness at the individual level. This robustness is particularly relevant in clinical contexts, where minimizing the risk of poorly performing stimulation protocols is essential. A key advantage of the group optimization method is that it eliminates the need to preselect a single ‘representative’ head model, a task that can be particularly challenging in anatomically diverse populations.

The correlation observed between the NERNI scores and some anatomical characteristics (i.e. the volume of the head tissues and the perimeters of the head along the reference coordinate planes) indicates that there is room to improve the results of group optimization approaches by selecting subjects in an available data pool with anatomical characteristics that predict the highest NERNI scores in the target population. Our results showed that models can be built to make these predictions based on the tested anatomical characteristics of the participants’ heads. Models that use only anatomical features that can be obtained without MRI are particularly interesting. This model performs worse than the more complex model, but our results still show some predictive power. It is important to note that this regression analysis is exploratory: while useful for guiding subject selection, the associations with downstream performance metrics like NERNI and ${E_n}$ may reflect complex, nonlinear interactions. A more fundamental future direction would be to investigate correlations between anatomical features and variations in the lead-field matrix itself, or its dominant components, providing a more mechanistic basis for predicting E-field behavior. Although technically challenging due to mesh standardization requirements, such approaches could further refine individualized montage design in MRI-limited contexts. Furthermore, our head models did not include additional tissue classes such as compact/spongy bone or brain vasculature, which are supported in newer segmentation pipelines (e.g. SimNIBS 4.x, (Puonti *et al* 2020)). While the absence of these tissues is unlikely to affect the main conclusions regarding the relative advantages of group-based optimization, it may alter the results of our anatomical feature analysis. More detailed tissue representations could influence local E-field distributions and may reveal additional or stronger anatomical predictors of optimization performance.

Future work should test whether choosing subjects for the group used in the optimization based on these model predictions can improve the results. Another potential avenue for exploration lies in extending the anatomical characteristics considered. Of particular relevance, given previous work, is the local tissue thickness (Opitz *et al* 2015, Mosayebi-Samani *et al* 2021), which was not explored here but may further increase the predictive power of these models.

This study focused primarily on NERNI, a more comprehensive metric that integrates both field intensity and spatial accuracy relative to the target. We included the average *E_n_*-field on the target ($\left\langle {{E_n}} \right\rangle $) as a secondary metric for comparison because it is widely reported in the tES literature and provides a more intuitive measure of the stimulation strength. However, $\left\langle {{E_n}} \right\rangle $ alone does not account for focality. Indeed, high $\left\langle {{E_n}} \right\rangle $ values can coincide with poor targeting performance if the E-field significantly spreads beyond the high-weight target regions, resulting in lower NERNI scores. The balance between focality and E-field intensity on the target is encoded in the selected weights and target ${E_n}$ value used in the optimization: a higher target ${E_n}$ and a higher ratio of weights on the target to off-target results in montages with higher $\left\langle {{E_n}} \right\rangle $, but also with worse focality.

The correlation between NERNI and stimulation effects has not yet been quantified, but several studies have reported positive effects of NERNI/ERNI-based optimizations: Fischer *et al* (2017), Kaye *et al* (2021), Sprugnoli *et al* (2021), Zhou *et al* (2021), Daoud *et al* (2022), Ruffini *et al* (2024). Furthermore, a linear relationship has been shown between performance on some tasks, such as dual-task error (Manor *et al* 2018) and $\left\langle {{E_n}} \right\rangle $ following stimulation of the lDLPFC (Salvador *et al* 2022). Given the linear relationship observed between NERNI and $\left\langle {{E_n}} \right\rangle $ in the optimized montages (see figure 6(a)), it is likely that NERNI also correlates with stimulation effects. However, the effectiveness of NERNI, hinges on the relevance of the target map for the specific application. It should be noted that group-based optimization can be performed regardless of the objective function of the optimization, and the methods reported in this paper are still valid.

This group-based approach was employed successfully in a trial on depression targeting the lDLPFC (Ruffini *et al* 2024), using a cohort of patients selected to have an age range matching that of the target population. Another variation of this approach is currently being tested, in which a group optimization based on a cohort of patients with similar demographic characteristics is first performed to obtain a common pool of electrodes for every subject, and then the currents are personalized either fully (NERNI minimization, using the lDLPFC as a target, as is currently being performed in the trial: NCT06821568), or by scaling them via correlations obtained from the head perimeters (more details in Mencarelli *et al* 2025, in prep, where the targets were selected due to their relevance in Alzheimer’s onset and progression). These applications highlight the potential of group-based optimization in real-world settings and emphasize the need for further refinement in selecting the most representative head models.

The fact that this study focused only on one target area should not be perceived as a limitation, as the general methodology is applicable to any target and, qualitatively, the results should not change. The hyper parameters used for the optimization (i.e. the weights and target En values) potentially have a stronger influence on the results. In particular, we observed that with a target En of 0.25 V m^−1^, the participant specific optimized protocols tended to use all available total injected currents, for the majority of cases. This would not be the case for lower target En values (for instance, 0.10 V m^−1^). While we did not directly test this regime, future work could investigate whether such conditions enhance the relative advantage of group-based over template-based protocols. The biggest advantage of lowering the target En would be to obtain a better focality of stimulation (i.e. restricting the montage to the target area) at the cost of average En on the target.

**Figure 10.**
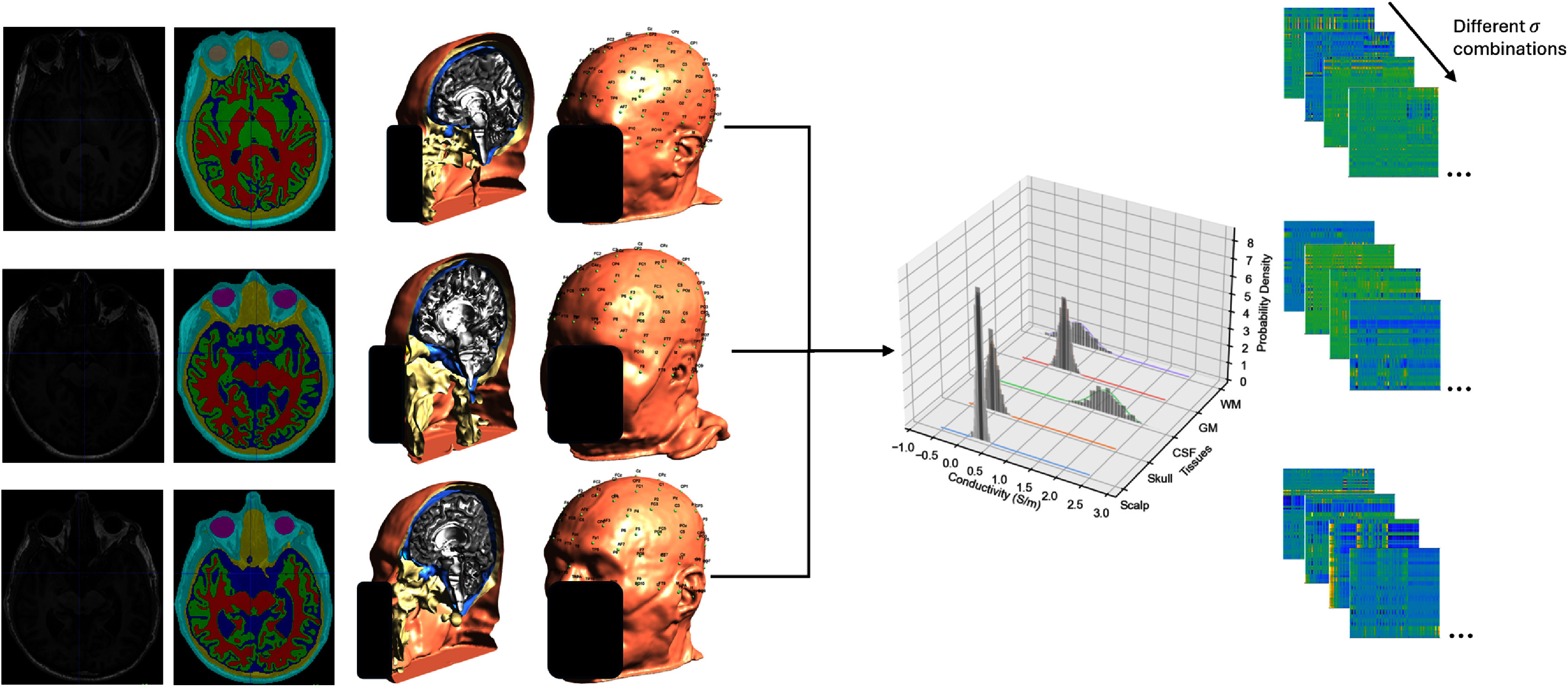
Extension of the group optimization framework to account for variability in tissue conductivity. In this approach, each participant’s head model is repeated multiple times with different combinations of tissue conductivities, sampled from known or assumed population distributions. This enables the optimization to incorporate uncertainty in conductivity values and better reflect inter-subject variability in biophysical properties. This extension can be used to build more robust protocols when individual tissue conductivities are not precisely known.

The results of the current study were based on a relatively small sample size. Future studies should focus on leveraging large databases with head structural MRIs suitable for biophysical modeling. This would increase the predictive power of the observed correlations and enable the development of strategies for stratification according to relevant anatomical/demographic features, which could mitigate the increased inter-subject variability typically associated with larger cohorts. Additionally, this study utilized standard conductivities for the different segmented tissues. As more data emerge showing considerable inter-subject variability in tissue conductivity and that such variability induces meaningful effects on E-field distribution (Saturnino *et al* 2019c, Antonakakis *et al* 2020), it is important to take this into account in the optimization strategies. As more information is available about the expected distribution of tissue conductivities across the population, this information can be considered by expanding the database with the same biophysical head models with different conductivity values for the different tissues (see figure 10).

## Data Availability

The data cannot be made publicly available upon publication because they are not available in a format that is sufficiently accessible or reusable by other researchers. The data that support the findings of this study are available upon reasonable request from the authors.

## Data Access Statement

The data that support the findings of this study are available from the corresponding author, R.S., upon reasonable request.
